# Evaluation of the BL-RED test and comparison with the β-LACTA test for rapid detection of resistance to third-generation cephalosporins in *Enterobacterales* on early culture of positive blood cultures

**DOI:** 10.1128/spectrum.02718-25

**Published:** 2026-02-04

**Authors:** Mehdi Bonnet, Yahia Benzerara, Maxime Danjean, Paul Louis Woerther, Christophe Rodriguez, Orlando Schilton, Nicolas Veziris, Alexandre Godmer, Gautier Pierrat

**Affiliations:** 1 Département de Bactériologie, Hopital Saint-Antoine, APHP.Sorbonne Université 37117, Paris, France; 2 Service de Bactériologie et Hygiène, CHU Henri Mondor, Assistance Publique-Hôpitaux de Paris, Créteil, France; 3 EA 7380 DYNAMYC, Université Paris-Est Créteil 27010 , Créteil, France; 4 Centre d’Immunologie et des Maladies Infectieuses, INSERM, U1135, Sorbonne Université 27063, Paris, France; Assistance Publique-Hopitaux de Paris Universite Paris Saclay, Clamart, France

**Keywords:** BL-RED test, BETA-LACTA test, rapid tests, cephalosporin, multidrug resistance, ESBL, Enterobacteriaceae

## Abstract

The dissemination of multidrug-resistant *Enterobacterales*, particularly those resistant to third-generation cephalosporins (3GC), poses a significant public health challenge. This study evaluated the performance of the Beta-Lactamase Rapid Electrochemical Detection (BL-RED) test (CORIS BioConcept, Belgium) alongside the β-LACTA test (Bio-Rad, France), another routinely used technique in our laboratory, for the rapid detection of 3GC-resistant *Enterobacterales* from 4 h subcultures of positive blood cultures. A total of 178 isolates were included: 55 clinical isolates with characterized resistance mechanism and 123 isolates from positive blood cultures prospectively collected between April and July 2023. Parallel testing with the BL-RED and β-LACTA tests was performed, and the results were compared to those of conventional antibiotic susceptibility testing by disk diffusion, following the 2024 EUCAST recommendations. The sensitivity, the specificity, the observed positive predictive value, and the observed negative predictive value for 3GC resistance detection in the prospective cohort were 74%, 100%, 100%, and 95%, respectively, for both the BL-RED and β-LACTA tests. Both tests demonstrated good performance in detecting 3GC-resistant *Enterobacterales*, particularly those expressing common Ambler class A beta-lactamases. However, they were less effective in detecting 3GC resistance due to the presence of other Ambler class families, notably overproduced chromosomal and plasmid-mediated Ambler class C beta-lactamases. The ease of use of these tests makes them suitable for routine microbiology laboratory testing. We propose a diagnostic algorithm combining the use of either the BL-RED and β-LACTA tests with that of MALDI-TOF MS in order to initiate targeted antibiotic therapy early.

**IMPORTANCE:**

Antimicrobial resistance, especially among *Enterobacterales* resistant to third-generation cephalosporins (3GC), represents a major public health threat. Delayed detection of these resistant pathogens can lead to inappropriate empirical antibiotic therapy, with risk of treatment failures and further spread of resistance. This study underscores the value of rapid diagnostic tools, specifically the BL-RED and β-LACTA tests, for the rapid identification of 3GC-resistant *Enterobacterales* directly from positive blood cultures. By enabling quicker therapeutic decision-making, these assays provide a practical solution for microbiology laboratories to enhance patient management and contribute to global efforts in mitigating the impact of antibiotic-resistant infections.

## INTRODUCTION

The spread of multidrug-resistant *Enterobacterales* represents a significant threat to global public health. According to the World Health Organization, *Enterobacterales* resistant to third-generation cephalosporins (3GC) and carbapenems are identified as critical pathogens for new antibiotics research and development (1). The increasing resistance to 3GC is not only due to the dissemination of plasmid-encoded extended-spectrum beta-lactamases (ESBL) but also the overexpression of chromosomal Ambler class C beta-lactamase (AmpC) cephalosporinases, plasmid-mediated AmpC cephalosporinases, and the emergence of carbapenemases (2). According to data from the European Center for Disease Prevention and Control (ECDC) published in 2021, the prevalence of 3GC resistance among clinical isolates of *Klebsiella pneumoniae* and *Escherichia coli* from invasive infections (blood and cerebrospinal fluid cultures) in France in 2021 was 25.4% and 8.3%, respectively (3). Bacteremia caused by ESBL-producing *Enterobacterales* is associated with increased treatment failure, higher mortality, and greater hospital costs (4, 5). Delayed initiation of effective antibiotic therapy for ESBL-producing *Enterobacterales* is a major risk factor for increased mortality (6). Consequently, rapidly optimizing antibiotic therapy based on pathogen identification and resistance profiling is a key objective of paramount importance. However, conventional microbiological methods require between 12 and 48 h for bacterial identification and antibiotic susceptibility testing. In recent years, rapid diagnostic tests have been developed to quickly identify causative microorganisms and their resistance phenotypes, especially to beta-lactam antibiotics, directly from early solid cultures of positive blood cultures (7). These techniques support antimicrobial stewardship by enabling prompt adaptation of effective treatment, thereby mitigating the medical and economic burden through antibiotic de-escalation and shorter hospital stays (8).

This study aimed to evaluate the performance of the Beta-Lactamase Rapid Electrochemical Detection (BL-RED) test (CORIS BioConcept, Belgium) alongside another colorimetric technique routinely used in our laboratory, the β-LACTA test (Bio-Rad, France), for detecting 3GC resistance through enzymatic hydrolysis in early subcultures of positive blood cultures. First, the tests were performed on a collection of *Enterobacterales* isolates from our laboratory with known resistance phenotypes and genotypes. Second, we performed the tests under real-life conditions on *Enterobacterales* isolates prospectively collected from positive blood cultures between April and July 2023. Finally, we propose an algorithm for rapidly optimizing antibiotic therapy for septic patients with positive blood cultures. This algorithm is based on MALDI-TOF MS identification combined with the results of the BL-RED or β-LACTA tests.

## MATERIALS AND METHODS

### Collection of *Enterobacterales* isolates with characterized resistance mechanisms

This collection includes 55 clinical strains isolated by the bacteriology department of Saint-Antoine Hospital in Paris, France, from samples taken from patients hospitalized in three Parisian hospitals: Tenon Hospital, Saint-Antoine Hospital, and Trousseau Hospital. The collection was stored at −20°C in a glycerol medium and thawed as needed. The species distribution was as follows: *Escherichia coli* (*n* = 23), *Enterobacter cloacae complex* (*n* = 9), *Citrobacter freundii* (*n* = 8), *Klebsiella pneumoniae* (*n* = 5), *Klebsiella oxytoca* (*n* = 2), *Citrobacter koseri* (*n* = 1), *Klebsiella aerogenes* (*n* = 2), *Hafnia alvei* (*n* = 1), *Proteus mirabilis* (*n* = 2), *Proteus vulgaris* (*n* = 1), and *Serratia marcescens* (*n* = 1).

All isolates were screened for the presence of major Ambler class A beta-lactamase genes (*bla*_TEM_, *bla*_SHV_, *bla*_CTX-M,_ and *bla*_IMI_) (9), class B carbapenemase genes (*bla*_NDM_, *bla*_VIM,_ and *bla*_IMP_) (10), class C cephalosporinase genes (*bla*_ACC_, *bla*_MOX_, *bla*_FOX_, *bla*_CMY_, *bla*_LAT_, *bla*_ACT-1,_ and *bla*_DHA_) (9), and class D oxacillinase gene (*bla*_OXA-48-like_) (10) using PCR (Table S1). Amplified products were sequenced by Sanger sequencing and analyzed using a dedicated database (11). The expressions of genes encoding carbapenemases or ESBL were confirmed using O.K.N.V.I RESIST-5 and RESIST CTX-M immunochromatographic tests (Table S1). The O.K.N.V.I RESIST-5 kit detected the following carbapenemases: *bla*_OXA-48-like_, *bla*_KPC_, *bla*_NDM_, *bla*_VIM_, and *bla*_IMP_. The RESIST CTX-M kit detected only CTX-M group 1 and 9 ESBLs.

The collection included strains exhibiting various beta-lactam resistance phenotypes: 3GC-susceptible (*n* = 27) and 3GC-resistant (*n* = 28). Among the 3GC-resistant strains, 23 were ESBL-producers, 3 were chromosomal hyperproducers or plasmid-mediated AmpC producers, and 8 were carbapenemase producers. The presence of ESBL, plasmid-mediated AmpC cephalosporinase, and carbapenemase genes was confirmed by specific PCR (Table S1).

For each isolate, 1 mL of a 0.5 McFarland suspension was inoculated into sterile aerobic blood culture bottles (BACT/ALERT FA Plus, Biomérieux, France). Blood cultures were incubated at 35°C ± 2°C for 16 h. Subcultures were grown on PolyViteX chocolate agar (Biomérieux, France) and incubated at 35°C ± 2°C for 4 h. Isolated colonies were identified by MALDI-TOF mass spectrometry (Bruker, USA), and antibiotic susceptibility test (AST) was performed.

### Prospectively evaluated positive blood cultures

Positive blood cultures were prospectively evaluated from patients between April and July 2023. To avoid redundancy, only one blood culture per bacteremia episode was included.

According to our laboratory’s routine practice, positive blood cultures flagged by the automated BACT/ALERT VIRTUO system (BioMérieux, France) before 11 a.m. were subcultured onto PolyViteX chocolate agar (BioMérieux, France) and incubated at 35°C ± 2°C for 4 h. After this short incubation period, the colonies were identified by mass spectrometry using the MALDI-TOF Biotyper system (Bruker Daltonics, Germany). Only species belonging to the Enterobacterales order were included in the study. Isolates with positive BL-RED and/or β-LACTA tests were further tested using RESIST CTX-M and O.K.N.V.I RESIST-5 immunochromatographic assays (CORIS BioConcept, Belgium), according to the manufacturer’s instructions. Additionally, isolates with positive BL-RED or β-LACTA tests, as well as those resistant to 3GC (excluding AmpC hyperproduction), were screened for beta-lactam resistance genes and clonal lineage by whole-genome sequencing (WGS).

### WGS and genotyping

Bacterial DNA was extracted from pure colonies using the QIAsymphony instrument (Qiagen, Germany). Whole genomes were sequenced with the NovaSeq6000 instrument using the DNA Prep library kit (Illumina, USA). Raw reads have been deposited in the European Nucleotide Archive (ENA) at EMBL-EBI under accession number PRJEB87371. They were trimmed using Trimmomatic v0.39 (12) and *de novo-* assembled using the SPAdes-based shovill algorithm (13). Assembly contiguity was assessed using Quast v5.2.0 (14) with a minimum N50 value of 100 kb and, genome contaminations were checked with checkM v1.2.2 (15) lineage workflow with a maximum threshold of 5%.

Multi-locus sequence typing (MLST) was performed using mlst (https://github.com/tseemann/mlst). The Warwick MLST scheme was used for *E. coli* isolates. Genomic diversity was assessed by core-genome MLST (cgMLST) using the chewBBACA pipeline (16) v3.3.2 and the relatedness threshold of 10 alleles (17) to filter out identical genomes. *Enterobacterales* resistance genes were screened using the Abricate pipeline v1.0.1 (https://github.com/tseemann/abricate) with the ResFinder database (18) with standard parameters of identity and coverage.

### β-LACTA

The β-LACTA test is based on the hydrolysis of a chromogenic cephalosporin analog substrate (HMRZ-86), which results in a color change from yellow to red within 15 min for a positive test result. In the absence of hydrolysis—and, consequently, a color change—the result is considered negative. A color change resulting in a final color other than red is considered uninterpretable. For this test, a 1 µL loop was used to sample colonies from the 4 h bacterial preculture. After 15 minutes of incubation at 35°C ±2°C (instead of the 18°C–30°C range specified by the manufacturer), the presence or absence of a color change was observed.

### RESIST CTX-M and O.K.N.V.I RESIST-5

RESIST CTX-M (CORIS BioConcept, Belgium) is an immunochromatographic test designed for the rapid detection of ESBL-producing strains that express CTX-M enzymes from groups 1 (including CTX-M-15) and 9 (including CTX-M-14). O.K.N.V.I RESIST-5 (CORIS BioConcept, Belgium) is an immunochromatographic assay for the rapid detection of strains producing OXA-48-like, KPC, NDM, VIM, and IMP carbapenemases.

### BL-RED

The BL-RED test is based on the presence of a 3GC-analog substrate present in the reagent, which undergoes hydrolysis in the presence of a 3GC-active beta-lactamase, generating an electro-conductive product. The resulting electrochemical signal is measured in nanoamperes (nA) using a disposable graphite electrode, with a positive threshold of 50 nA. We followed the manufacturer’s instructions (Fig. S1) to perform this test, except we incubated the reaction mixture for 15 min instead of the specified 10 min to align with the β-LACTA test’s incubation conditions.

### Gold-standard comparator method

The gold standard for determining resistance to 3GC is the disk diffusion susceptibility testing method, which is performed according to the 2024 EUCAST recommendations. SIRscan disks containing 10 µg of ceftazidime and 5 µg of cefotaxime (I2A, France) were used for this purpose (19). Strains were classified as 3GC-resistant if the inhibition zone diameters were <19 mm for ceftazidime and <17 mm for cefotaxime. ESBL detection was determined by the presence of synergy between ceftazidime and cefotaxime disks and a 20/10 µg amoxicillin/clavulanic acid SIRscan disk (i2a, France). All ceftazidime- and/or cefotaxime-resistant strains were retested using the diffusion method on Mueller-Hinton medium supplemented with 250 mg/mL cloxacillin (MHC250) to detect possible cephalosporinase overproduction. An increase of 5 mm in the inhibition diameter around the 3GC disks on the MHC250 medium, compared to the non-supplemented Mueller-Hinton medium, confirmed the overproduction of cephalosporinase (20). We defined “low-level penicillinase” as resistance to amino-, carboxy-, and ureido-penicillins, with preserved susceptibility to penicillin/beta-lactamase inhibitor combinations. We categorized “high-level penicillinase” as resistance to amino-, carboxy-, and ureido-penicillins as well as to penicillin/beta-lactamase inhibitor combinations (with the exception of piperacillin-tazobactam).

### Statistical analysis

The sensitivity, specificity, positive predictive value (PPV), and negative predictive value (NPV) observed for the BL-RED and the β-LACTA tests were calculated and compared with those of the gold-standard method. A negative BL-RED / β-LACTA test result for a 3GC-susceptible isolate was considered a true negative, while a positive result for a 3GC-resistant isolate was considered a true positive. Conversely, a negative result for a 3GC-resistant isolate was considered a false negative, and a positive result for a 3GC-susceptible isolate was considered a false positive.

The exact McNemar’s test was used to evaluate the agreement between each test and the gold standard, accounting for small cell counts in the contingency table.

Statistical analysis was performed using R (v4.4.3) with the caret package (21).

## RESULTS

### Collection of *Enterobacterales* isolates with characterized resistance mechanism

Among the 55 isolates in our collection, 27 were susceptible to 3GC according to the AST reference method (Table S1). Twenty-three of the 3GC-susceptible isolates were wild-type, expressed low or high levels of penicillinase, or expressed their chromosomal cephalosporinase, AmpC. The remaining four strains carried the blaOXA-48 gene, and the O.K.N.V.I RESIST-5 immunochromatographic test detected the expressions of three of these. All of these isolates tested negative for BL-RED, β-LACTA, and RESIST CTX-M.

Twenty-eight strains were 3GC-resistant. Of these, 24 tested positive with both the BL-RED and β-LACTA tests. Three 3GC-resistant *Enterobacterales* strains, confirmed to overexpress chromosomal cephalosporinase via growth on Mueller-Hinton agar with 250 mg/L cloxacillin, tested negative with both BL-RED and β-LACTA assays. A single discrepancy was observed with a TEM-3 ESBL-producing *Escherichia coli* strain with a positive β-LACTA test and a negative BL-RED test. A total of 20 out of 28 strains, all positive with β-LACTA and BL-RED tests, were also positive with the RESIST CTX-M test (15 belonged to group 1 and 5 to group 9). Of the strains tested positive for BL-RED and β-LACTA, eight strains carried at least one carbapenemase-encoding gene with expression confirmed by the O.K.N.V.I RESIST-5 test for seven strains: one strain produced an NDM enzyme, five strains co-produced an OXA-48 and a CTX-M group 1 (*n* = 4) or 9 (*n* = 1) enzymes, and two strains co-produced either NDM and OXA-48 enzymes or VIM and an ESBL SHV-5.

In our sample, the sensitivity of the BL-RED and β-LACTA tests for detecting 3GC resistance *in vitro* was 86% and 89%, respectively; the specificity was 100% for both tests. The concordance and Cohen’s kappa coefficient between the β- LACTA and BL- RED tests were high, at 98% and 0.96, respectively. The observed PPV was 100% for both tests, and the observed NPV was 87% and 90%, respectively. It is noteworthy that 23 of the 24 strains that were detected as positive with the BL-RED test produced SHV- or CTX-M-type ESBL enzyme. With regard to the detection performance of carbapenemases, the presence of insufficient numbers of VIM and NDM enzymes precluded the drawing of any conclusions. However, 10 strains were observed to produce OXA-48. A total of 6 out of 10 strains that co-produced an ESBL-type (*n* = 5) or a VIM-type carbapenemase (*n* = 1) were detected as positive with the BL-RED test. In contrast, the remaining 4 out of 10 strains did not co-produce ESBL, demonstrated sensitivity to 3GCs *in vitro*, and were identified as negative with the BL-RED test.

### Prospective evaluation of positive blood cultures

A total of 123 blood cultures were prospectively included in the study. The distribution of bacterial species isolated and identified by MALDI-TOF mass spectrometry (Bruker, USA) and their resistance phenotype determined by AST are summarized in Table 1.

**TABLE 1:** Species of *Enterobacterales* isolated from positive blood cultures and their phenotypic resistance mechanisms^*a*^

Species	Wild-type	Low-level penicillinase	High-level penicillinase	Piperacillin-tazobactam-resistant^*b*^	Overproduced AmpC	Plasmid-mediated AmpC	ESBL	Total
*Citrobacter koseri*	1							1
*Enterobacter cloacae complex*	5	1		1	2		2	11
*Escherichia coli*	26	2	18	14		2	7	69
*Klebsiella oxytoca*	3							3
*Klebsiella pneumoniae*	13		1	2		1	4	21
*Klebsiella variicola*	1							1
*Klebsiella oxytoca*	1							1
*Morganella morganii*	2							2
*Proteus mirabilis*	2	1						3
*Salmonella enterica* subs. *Enterica*	1	1	1					3
*Serratia* sp.	7							7
Total	62	5	20	17	2	3	13	123

^*a*^ AmpC, cephalosporinase (Ambler class C enzyme); ESBL, extended-spectrum beta-lactamases.

^*b*^ except strains producing ESBL, carbapenemases, or overproducing AmpC.

Thirteen isolates were found to be positive for both the BL-RED and β-LACTA tests. These isolates exhibited ESBL production according to the phenotypic reference method. The mean BL-RED assay value for positive results was 3,057.70 nA (IC95% = [1,654–4,461]). WGS of these isolates (Table S2) revealed the presence of a CTX-M group 9 gene (CTX-M-9,-14, -18,-24, and −27) in 4 isolates. In addition, 9 isolates carried a CTX-M group 1 gene (CTX-M-1, -3, and −15). The identity and coverage percentages for all genes exceeded 99%. Of these, only 8 exhibited a positive result for the RESIST CTX-M test, specifically 5 with CTX-M-15 and 3 with CTX-M-14. Consequently, 4 isolates producing CTX-M-15 and 1 isolate producing CTX-M-27 yielded false-negative results with the RESIST CTX-M test.

A total of 109 isolates were found to be negative for the BL-RED and β-LACTA tests. All BL-RED values were recorded as 0.00 nA. Among these, 104 were determined to be 3GC-susceptible and true negative according to the reference method, comprising 62 wild-type isolates, 25 isolates producing low- or high-level penicillinases, and 17 isolates exhibiting piperacillin-tazobactam resistance (not ESBL, carbapenemases, nor AmpC), potentially due to inhibitor-resistant penicillinases or class D oxacillinases. Conversely, 5 isolates were found to be resistant to 3GC; of those, 2 isolates from the *Enterobacter cloacae* complex exhibited chromosomal AmpC overproduction and 3 isolates produced plasmid-mediated DHA-1 cephalosporinase (Table S3). These isolates were designated as false negatives.

Core-genome MLST analysis ruled out clonality among the isolates in this study, with the exception of two *E. coli* strains, which belonged to the same ST131 lineage and displayed an allelic distance of 1. Consequently, they were therefore regarded as being genomically identical according to the predefined threshold of ≤10 alleles. This finding suggests the possibility of clonality, particularly given that the two patients from whom the samples were obtained were admitted to the same department 2 months apart. Therefore, one of the two strains was excluded from further analysis.

The sensitivity and specificity of the BL-RED and β-LACTA tests were 72% and 100%, respectively. The observed PPV and the observed NPV were 100% and 95%, respectively, with an accuracy of 96% for both tests. These results are summarized in Table 2.

**TABLE 2:** Performance of the BL-RED and β-LACTA tests for the detection of 3GC-resistant *Enterobacterales* isolates from subcultures of positive blood cultures prospectively evaluated (real-life conditions)^*a*^

Method	Result	3GC		Sensitivity	Specificity	PPV^*b*^	NPV^*c*^	Accuracy (IC 95%)
		Resistant (*n* = 18)	Susceptible (*n* = 104)					
BL-RED	Positive	13	0	72%	100%	100%	95%	96% (0.9076, 0.9824)
	Negative	5	104					
β-LACTA	Positive	13	0	72%	100%	100%	95%	96% (0.9076, 0.9824)
	Negative	5	104					

^*a*^ 3GC, third-generation cephalosporin.

^*b*^ PPV, observed positive predictive value.

^*c*^ NPV, observed negative predictive value.

## DISCUSSION

The spread of ESBL- and carbapenemase-producing *Enterobacterales* is a serious public health concern worldwide. Resistance to 3GC in critical infections, such as bloodstream infections, can lead to an inappropriate first-line antibiotic therapy with a known impact on mortality (5). Therefore, the rapid identification of 3GC-resistant *Enterobacterales* in the clinical microbiology laboratory is of utmost importance.

In this study, we evaluated the performance of the BL-RED and β-LACTA tests compared to our gold-standard method, both of which are rapid and easy to use in the microbiology laboratory. Regarding our collection of *Enterobacterales* isolates with characterized resistance mechanisms, both tests showed similar performance in detecting 3GC resistance, with a specificity of 100% and sensitivities of 86% and 89%, respectively. Both tests had an observed PPV of 100% and observed NPVs of 87% and 90%, respectively. The difference in sensitivity was attributed to the failure of the BL-RED test to detect 3GC resistance in a TEM-3 ESBL-producing strain. Regarding the isolates from the prospectively evaluated positive blood cultures, the performance of both tests was identical, with a sensitivity of 72% and specificity of 100%, as well as an observed PPV and NPV of 100% and 95%, respectively. Both tests demonstrated promising performance in detecting 3GC resistance due to ESBL production, suggesting their potential utility in guiding early therapeutic decisions, particularly switching from 3GC to carbapenems. However, 3GC resistance due to overproduced chromosomal AmpC or plasmid-mediated AmpC was not detected by either test. These results are consistent with those of previous studies evaluating the β-LACTA (22–24) and the BL-RED (25–27) tests. WGS of 3GC-resistant isolates from prospectively included blood cultures confirmed the ability of both tests to detect 3GC resistance due to ESBL, but not AmpC, particularly plasmid-mediated AmpC (e.g., DHA-1 in our study). Furthermore, cgMLST revealed that these isolates belonged to diverse clonal lineages, mitigating potential confounding factors.

Our study has some limitations, including its monocentric design and the limited number of chromosomal AmpC (group 3 *Enterobacterales*) or plasmid-mediated AmpC-producing isolates tested. The small number of 3GC-resistant isolates with other enzymatic mechanisms, such as hyperproduction of chromosomal enzymes OXY and SHV, extended-spectrum OXA, or carbapenemases (particularly Ambler classes A and B), is also a limitation.

Due to their principle based on the hydrolysis of 3GC analogs, neither test can detect 3GC resistance due to impermeability or efflux pumps, although these mechanisms are less common than beta-lactamase production in *Enterobacterales*. While both tests showed similar performance, the BL-RED test offers the advantage of an output value, enabling traceability and minimizing uninterpretable results or operator-related errors that can occur with the β-LACTA test.

To rapidly optimize antibiotic treatment for septic patients (within 5 h blood culture positivity), we propose a simple algorithm for positive blood cultures based on MALDI-TOF MS identification and the BL-RED or β-LACTA test for detecting 3GC-resistant *Enterobacterales* (Fig. S2). In case of a negative test, 3GC therapy is recommended, with early adaptation to a fourth-generation cephalosporin, such as cefepime, if a natural AmpC producer (*Enterobacterales* group 3) is identified by MALDI-TOF MS. A positive test could warrant RESIST CTX-M and O.K.N.V.I RESIST-5 immunochromatographic tests, particularly to guide carbapenem use if O.K.N.V.I RESIST-5 is negative and to implement patient isolation when either test is positive. Therapeutic adaptation based on AST is recommended the following day.

A prospective study is warranted to evaluate the algorithm’s impact on antibiotic therapy decisions (escalation or de-escalation). Previous studies evaluating the β-LACTA test have shown mixed results, demonstrating impact on early antibiotic therapy adaptation and/or patient isolation (8, 28) or no impact (29).

In conclusion, the BL-RED test, as well as the β-LACTA test, demonstrated good performance in detecting 3GC-resistant *Enterobacterales* from early subcultures of positive blood cultures, particularly with a 100% PPV for both tests, especially for common Ambler class A beta-lactamases responsible for 3GC resistance. However, the PPV and NPV are prevalence-dependent, necessitating evaluation in diverse populations. Both tests are less suitable for detecting 3GC resistance due to other Ambler class families and mechanisms. Their ease of use makes them suitable for routine microbiology laboratories. By reducing the detection time of 3GC resistance compared to conventional methods, they can help clinicians in guiding early and appropriate antibiotic therapy for septic patients, potentially improving their prognosis.

## SUPPLEMENTAL MATERIAL

Fig. S1 spectrum.02718-25-s0001.tiff Instruction of use of the BL-REDTM.

Fig. S2 spectrum.02718-25-s0002.tiff Algorithm proposed for the management of *Enterobacterales* positive-blood cultures in the routine microbiology laboratory.

Table S1 spectrum.02718-25-s0003.xlsx Phenotypic and genotypic characteristics of β-lactamase-producing strains (PCR and rapid test results).

Table S2 spectrum.02718-25-s0004.xlsx Correlation data between phenotypic tests (antibiotic susceptibility, synergy) and genotypic resistance profiles.

Table S3 spectrum.02718-25-s0005.xlsx Molecular profiles and sequence types (MLST) of the studied isolates.

ASM does not own the copyrights to Supplemental Material that may be linked to, or accessed through, an article. The authors have granted ASM a non-exclusive, world-wide license to publish the Supplemental Material files. Please contact the corresponding author directly for reuse.
