# Supplementary figures and images for: Evaluation of the BL-RED test and comparison with the β-LACTA test for rapid detection of resistance to third-generation cephalosporins in Enterobacterales on early culture of positive blood cultures
Source: Microbiol Spectr. 2026 Feb 4;14(3):e02718-25. doi: 10.1128/spectrum.02718-25 (PMC12955405; doi:10.1128/spectrum.02718-25)

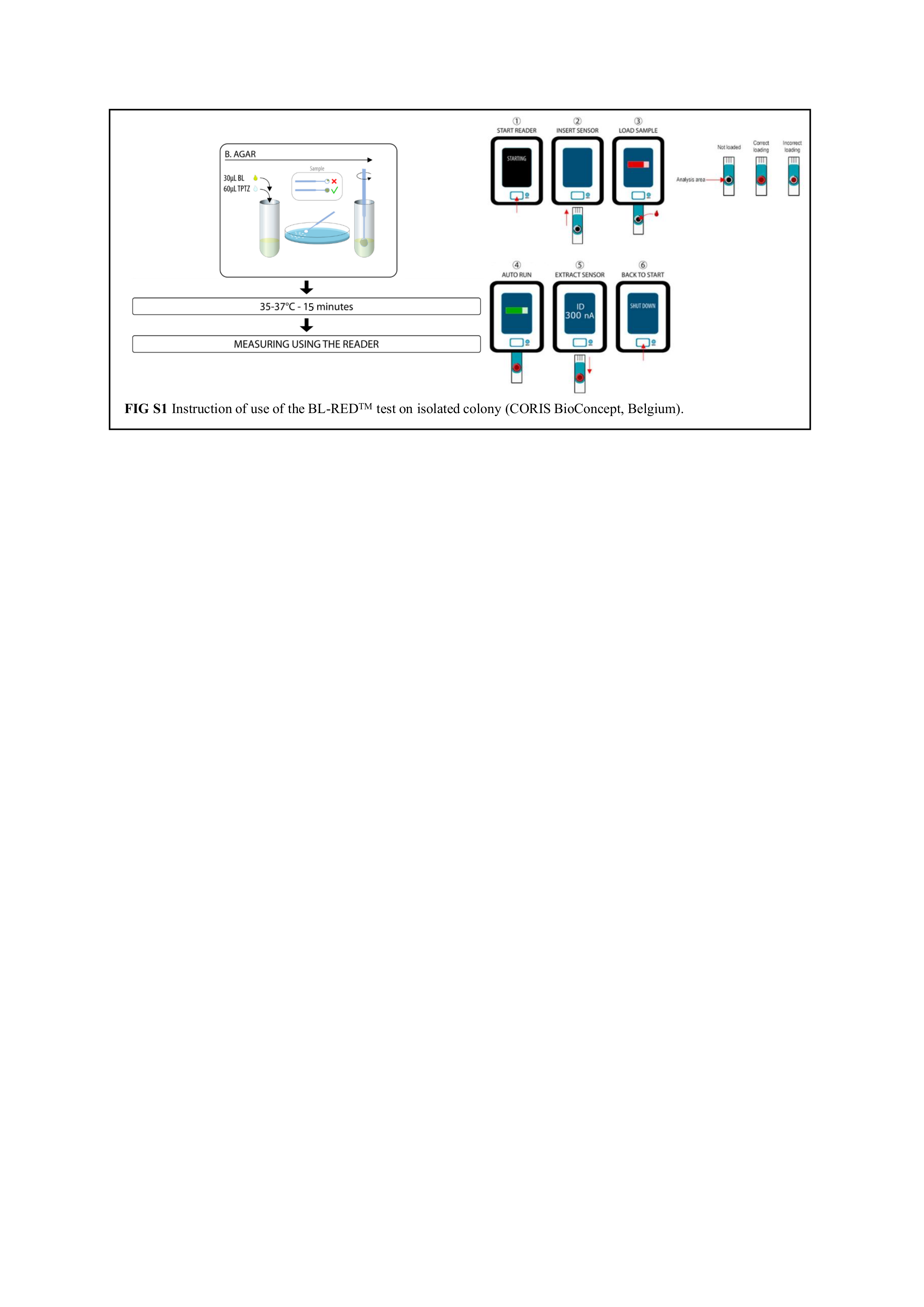

Supplement: Fig. S1 — Instruction of use of the BL-REDTM. [file spectrum.02718-25-s0001.tiff]

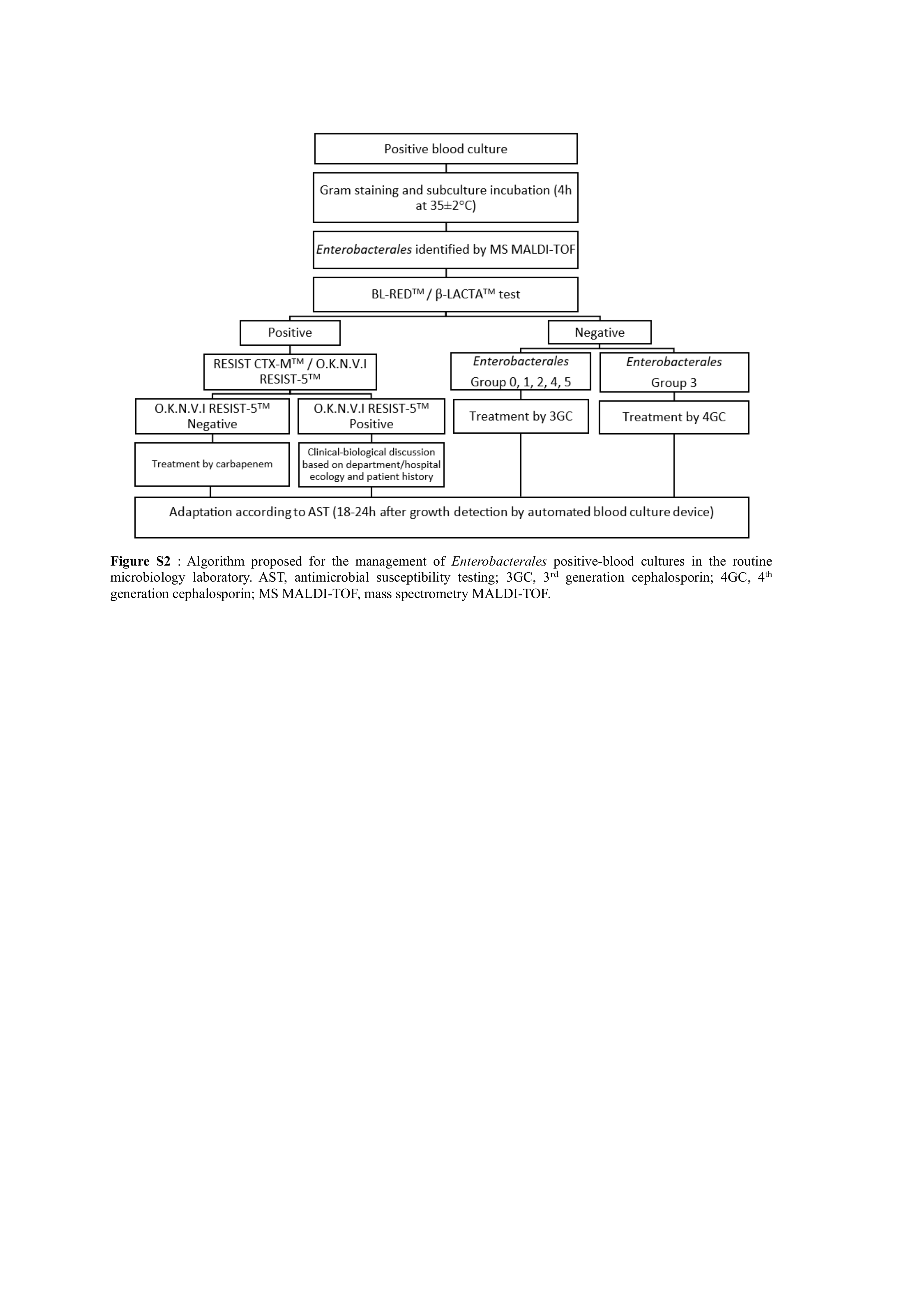

Supplement: Fig. S2 — Algorithm proposed for the management of Enterobacterales positive-blood cultures in the routine microbiology laboratory. [file spectrum.02718-25-s0002.tiff]
